# The assessment of mesenchymal stem cells therapy in acute on chronic liver failure and chronic liver disease: a systematic review and meta-analysis of randomized controlled clinical trials

**DOI:** 10.1186/s13287-022-02882-4

**Published:** 2022-05-16

**Authors:** Yuwei Liu, Yutong Dong, Xiaojing Wu, Xiaotong Xu, Junqi Niu

**Affiliations:** 1 Department of Hepatology, First Bethune Hospital of Jilin University, No. 71 XinMin Street, Changchun, 130021 Jilin People’s Republic of China; 2 Center for Pathogen Biology and Infectious Disease, First Bethune Hospital of Jilin University, Changchun, 130021 Jilin People’s Republic of China

**Keywords:** Mesenchymal stem cells, Liver disease, Efficacy, Safety, Meta-analysis

## Abstract

**Background:**

Mesenchymal stem cells (MSCs) therapy is showing potential therapeutic effects on liver function improvement in patients with chronic liver disease; however, the consensus on efficacy and safety of MSCs has not been reached.

**Methods:**

We performed this systematic review and meta-analysis of randomized controlled trials (RCTs) to evaluate the efficacy and safety of MSCs therapy for patients with chronic liver disease. A detailed search of the Cochrane Library, MEDLINE, Web of Science, and EMBASE databases was conducted to find studies published prior to September 15, 2021. The outcome measures were survival rate, model of end-stage liver disease (MELD) score, albumin, total bilirubin, coagulation function, and aminotransferase.

**Results:**

A literature search resulted in 892 citations. Of these, 12 studies met the inclusion criteria. It was found that compared with conventional treatment, MSCs therapy was associated with improved liver function including the MELD score, albumin levels, and coagulation function. However, it had no obvious beneficial effects on survival rate and aminotransferase levels. Subgroup analyses indicated that MSCs therapy had therapeutic effects on patients with both acute on chronic liver failure (ACLF) and cirrhosis. BM-MSCs and UC-MSCs treatment had similar efficacy to improve liver function. The effectiveness varied slightly between the peripheral intravenous injection and hepatic arterial injection. Five studies reported that the only adverse event of the MSCs therapy was fever, and no serious adverse events and side effects were reported. Analysis on clinical symptoms showed that encephalopathy and gastrointestinal hemorrhage events were reduced after MSCs therapy.

**Conclusions:**

In conclusion, this study suggested that MSCs therapy could be a potential therapeutic alternative for patients with chronic liver disease in clinical practice.

**Supplementary Information:**

The online version contains supplementary material available at 10.1186/s13287-022-02882-4.

## Introduction

In recent decades, mesenchymal stem cells (MSCs) have emerged the most promising treatment of chronic liver diseases. MSCs can self-renew and differentiate into various cell types including hepatocytes [1]. MSCs can work as seed cells to repair or replace impaired and diseased tissues and organs, which can provide a novel therapeutic approach for various refractory diseases. Studies from animal models have shown that MSCs treatment can ameliorate liver fibrosis [2, 3], improve liver function, alleviate liver injury [4, 5] and reverse fulminant hepatic failure [6, 7]. Some clinical studies also suggested that the infusion of MSCs can improve liver function and alleviate related complications in patients with liver cirrhosis and liver failure [8–10]. Therefore, MSCs have attracted increased attention in the treatment of liver diseases.

MSCs are mostly derived from bone marrow which can also be isolated from other tissues and organs such as umbilical cord, peripheral blood and adipose tissue. In the initial clinical practice, autologous bone marrow-derived MSCs (BM-MSCs) were the most frequently investigated for the treatment of liver disease. In clinical trials, autologous BM-MSC infusion has been confirmed to be safe and effective in the short term, but long-term outcomes remain unsatisfactory [11]. Possible reasons might be impaired function of autologous MSCs due to advanced age [12, 13] and self-disease condition [14]. Allogeneic BM-MSCs treatment has potential advantages and might be free from the limitations of autologous MSC treatment. For example, the preparation period of allogeneic BM-MSCs is shorter and the treatment delays can be avoided compared with autologous BM-MSCs. Moreover, allogeneic BM-MSCs could be obtained from young, healthy donors and have advantages in proliferation, differentiation, cytokine production, or other desired properties. Clinical studies have found that allogeneic BM-MSCs are safe and feasible for treatment of patients with liver cirrhosis [15] and acute on chronic liver failure (ACLF) [10]. Recently, accumulated researches have indicated that umbilical cord-derived MSCs (UC-MSCs) transplantation is an ideal therapy alternative in different liver diseases. UC-MSCs can be obtained in large quantities from the discarded umbilical cord to achieve mass production, and the application of UC-MSCs has no additional invasive operation for both donors and recipients. Another distinct advantage is the decreased alloreactivity due to a low expression of class I and class II human leukocyte antigen [16]. Clinical studies have shown that UC-MSCs infusion significantly improved liver function in patients with decompensated liver cirrhosis [17] and increased the survival rates of patients with ACLF [8, 13, 18, 19].

Although a large number of researches including randomized controlled clinical trials (RCT) have been carried out to explore the effect of MSCs treatment on liver diseases, the research schemes and evaluation indexes of different studies were inconsistent. Moreover, although there have been several meta-analyses of stem cell therapy for chronic liver disease, few have investigated MSCs therapy based on RCTs and analyzed the influence of different factors on the therapeutic effects in detail. Thus, we conducted this systematical review and meta-analysis of all currently available RCTs to assess the therapeutic efficacy and safety of MSCs treatment on chronic liver disease.

## Methods

### Search strategy

This study was performed according to the Preferred Reporting Items for Systematic Reviews and Meta-analyses statement. The Cochrane Library, MEDLINE (PubMed), Web of Science, and Ovid EMBASE were searched in detail to find studies published prior to September 15, 2021. The research focus comprised the terms (“mesenchymal Stem Cell” OR “mesenchymal stromal cell”) AND (“liver disease” OR “cirrhosis” OR “liver failure” OR “hepatic disease”). Mesh terms and free words were combined to search in each database. Manual searches were performed based on electronic searches as a supplement.

### Eligibility criteria

Two authors (YL and YD) independently assessed studies for inclusion by screening title and abstract. The inclusion criteria were: (1) study design: RCTs; (2) study population: patients diagnosed with chronic liver disease; (3) experiment group: patients received mesenchymal stem cells therapy; and (4) control group: patients received conventional therapy. The exclusion criteria included: (1) studies that did not provide clinical data or were impossible to estimate the clinical data; and (2) review articles, case reports, letters, editorials, nonhuman studies and duplicate studies.

### Data extraction

Two researchers (YL and YD), respectively, screened the whole text and extracted data from each study title. Any disagreements were resolved through discussion with a third reviewer (XW). The following data were collected when available: (1) study characteristic: publication year, first author, research area; (2) study patient characteristics: number of enrolled patients, type of liver disease, cause of liver disease, follow-up time; (3) mesenchymal stem cells: cell type, cell dosage, times of treatment, administration route; (4) study outcomes: the result of survival rate, adverse events and clinical symptoms, model for end-stage liver disease (MELD) score, albumin (ALB), total bilirubin (TBIL), coagulation function (prothrombin activity (PTA) and international normalized ratio (INR)) and transaminase (alanine aminotransferase (ALT) and aspartate aminotransferase(AST)) at different follow-up time point. For the articles that did not show the data directly, we tried to digitize the graphs and extract the data points using Engauge Digitizer software (version 5.1, http://digitizer.sourceforge.net).

### Quality assessment

Quality assessment was performed using Review Manager (version 5.3) according to the recommendations from the Cochrane Collaboration [20]. The bias risk assessment tool recommended by Cochrane was used to assess the quality of all enrolled studies. Each item of studies was judged as high, low or unclear risk of bias. Two researchers (YL and YD) independently evaluated the quality of the articles and the risk of bias, and a third author (XW) settled any subsequent disagreements.

### Statistical analysis

Based on the enrolled studies, standardized mean difference (SMD) or odds ratio (OR) with 95% confidence interval (CI) values was calculated using different effect models according to heterogeneity. The heterogeneity was calculated using Cochrane Q test (P heterogeneity) and *I*^2^ statistic, in which *P* < 0.1 in the *Q* statistic or *I*^2^ statistic > 50% was used to indicate at least moderate statistical heterogeneity. Sensitivity analyses were conducted by moving one study at a time to determine potential sources of heterogeneity. Subgroup analyses were conducted to explore the potential influence factors. Funnel plot, Egger’s and Begg’s tests were conducted to examine the existence of publication bias. All statistical analyses were performed using STATA software (version 15.0). A *P* value < 0.05 was considered statistically significant.

## Results

### Search results

**Fig. 1:**
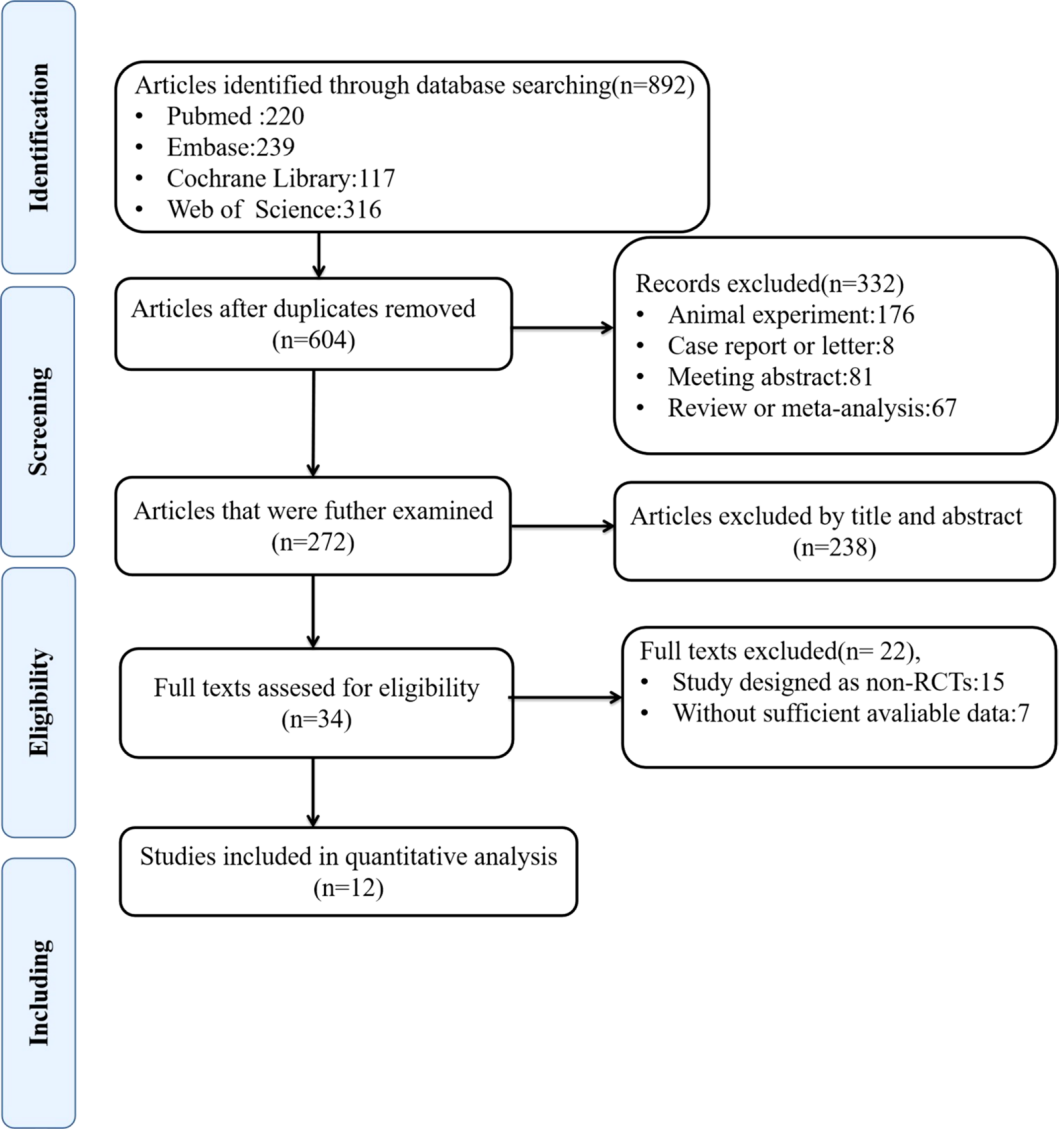
Flowchart of search strategy and study selection

Figure 1 illustrates the flowchart of the literature retrieval and screening procedures. Initial retrieve produced 892 articles. Two hundred and eighty-eight articles were excluded due to duplication. One hundred and seventy-six articles about the animal experiment, 8 case reports or letters, 67 reviews or meta-analyses, and 81 meeting abstracts were excluded. Two hundred and thirty-eight articles were excluded after reviewing their titles and abstracts, and 34 studies were further reviewed. Twenty-two studies were not included for the following reasons: (1) Sufficient data were not available; (2) the study did not use an RCT design. Finally, 12 studies [8–11, 19, 21–27] were included in the present meta-analysis.

### Study characteristics

The characteristics of the12 included studies are presented in Table 1. These studies were published between 2011 and 2021 from China (*n* = 7), Egypt (*n* = 2), South Korea (*n* = 1), Brazil (*n* = 1) and Iran (*n* = 1). A total of 846 patients were included, with 411 patients receiving MSCs therapy and 435 patients undergoing traditional supportive therapy. The studies included patients with cirrhosis (*n* = 6) and ACLF (*n* = 6). MSCs were derived from the bone marrow (BM-MSCs; *n* = 8) and umbilical cord (UC-MSCs; *n* = 4), 6 of which involve autologous transplants and 6 involve allogeneic transplants. MSCs were delivered through intravenous injection (*n* = 9) and hepatic arterial injection (*n* = 3). A single cell injection was adopted in 5studies, multiple cell injections in 6 studies, and both (single and multiple injections) in 1 study.

**Table 1:** Characteristics of the included randomized controlled trials

Year	Author	Country	Liver disease	Disease etiology	Cell type	Cell dosage	Times of treatment	Administration route	Patient number		Median age		Follow-up time	Adverse event
									Exp group	Con group	Exp group	Con group		
2021	Shi [27]	China	Decompensated cirrhosis	HBV	Allogeneic UC-MSCs	1 × 10^6^ /kg	Three	Intravenous injection	108	111	47	48	75 month	Fever
2021	Schacher [26]	Brazil	ACLF	Alcohol, HCV, HBV, NASH	Allogeneic BM-MSCs	1 × 10^6^/kg	Two	Intravenous injection	4	5	55.8	53.4	90 day	No
2019	Xu [19]	China	ACLF	HBV	Allogeneic UC-MSCs	1 × 10^5^/kg	Four	Intravenous injection	30	30	40.67	44.67	48 week	Fever
2017	Lin [10]	China	ACLF	HBV	Allogeneic BM-MSCs	(1.0–10) × 10^5^/kg	Four	Intravenous injection	56	54	40	42.8	24 week	Fever
2016	Suk [25]	South Korea	Decompensated Cirrhosis	Alcohol	Autologous BM-MSCs	5 × 10^7^/kg	One	Hepatic arterial injection	18	18	53.1	53.7	12 month	Fever
2016	Suk [25]	South Korea	Decompensated Cirrhosis	Alcohol	Autologous BM-MSCs	6 × 10^7^/kg	Two	Hepatic arterial injection	19	18	54.4	53.7	12 month	Fever
2014	Xu [24]	China	Cirrhosis	HBV	Autologous BM-MSCs	(0.75 ± 0.5) × 10^6^	One	Hepatic arterial injection	20	19	44	45	24 week	Fever
2014	Salama [9]	Egypt	Decompensated cirrhosis	HCV	Autologous BM-MSCs	1 × 10^6^/kg	One	Intravenous injection	20	20	50.27	50.9	6 month	No
2013	Mohamadnejad [23]	Iran	Decompensated cirrhosis	PBC, HBV, HCV, AIH	Autologous BM-MSCs	(1.2–2.95) × 10^8^	One	Intravenous injection	14	11	43.1	34.6	12 month	NA
2012	Zhang [8]	China	Decompensated cirrhosis	HBV	Allogeneic UC-MSCs	0.5 × 10^6^/kg	Three	Intravenous injection	30	15	48	47	48 week	No
2012	Shi [22]	China	ACLF	HBV	Allogeneic UC-MSCs	0.5 × 10^7^/kg	Three	Intravenous injection	24	19	40	45	48 week	No
2012	El-Ansary [21]	Egypt	Cirrhosis	HCV	Autologous BM-MSCs	1 × 10^6^/kg	One	Intravenous injection	15	10	48	51.6	6 month	NA
2011	Peng [11]	China	ACLF	HBV	Autologous BM-MSCs	(3.4 ± 3.8) × 10^8^	One	Hepatic arterial injection	53	105	42.19	42.2	192 week	No

ACLF, acute on chronic liver disease; HBV, hepatitis B virus; HCV, hepatitis C virus; NASH, nonalcoholic liver disease; PBC, primary biliary cholangitis; AIH, autoimmune hepatitis; BM-MSCs, bone marrow-derived mesenchymal stem cells; UC-MSCs, umbilical cord-derived mesenchymal stem cells; Exp,experiment; Con, control

### Quality assessment of included studies

**Fig. 2:**
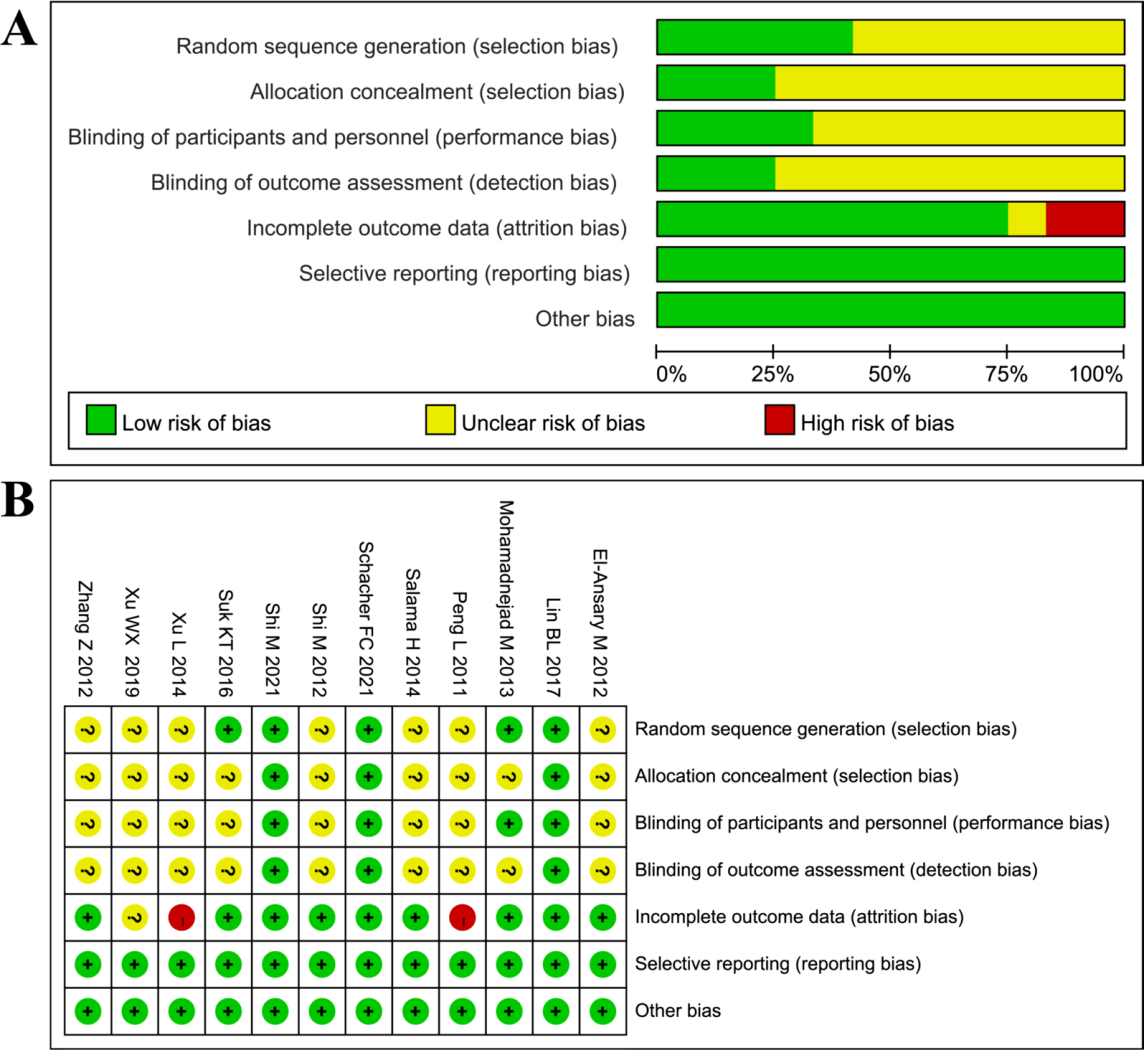
Risk of bias assessment. **A** Graph of the risk of bias for the included studies, **B** graph of the risk of bias summary for the included studies

Figure 2 presents the outcome of the quality assessment. There were 3 high-quality studies, 7 moderate-quality studies and 2 low-quality studies. The bias mainly came from a lack of random sequence generation, allocation concealment and blinding description. Two studies [11, 24] were considered to be high risk in attrition bias because of the data loss of patients during follow-up.

### Survival rate

**Fig. 3:**
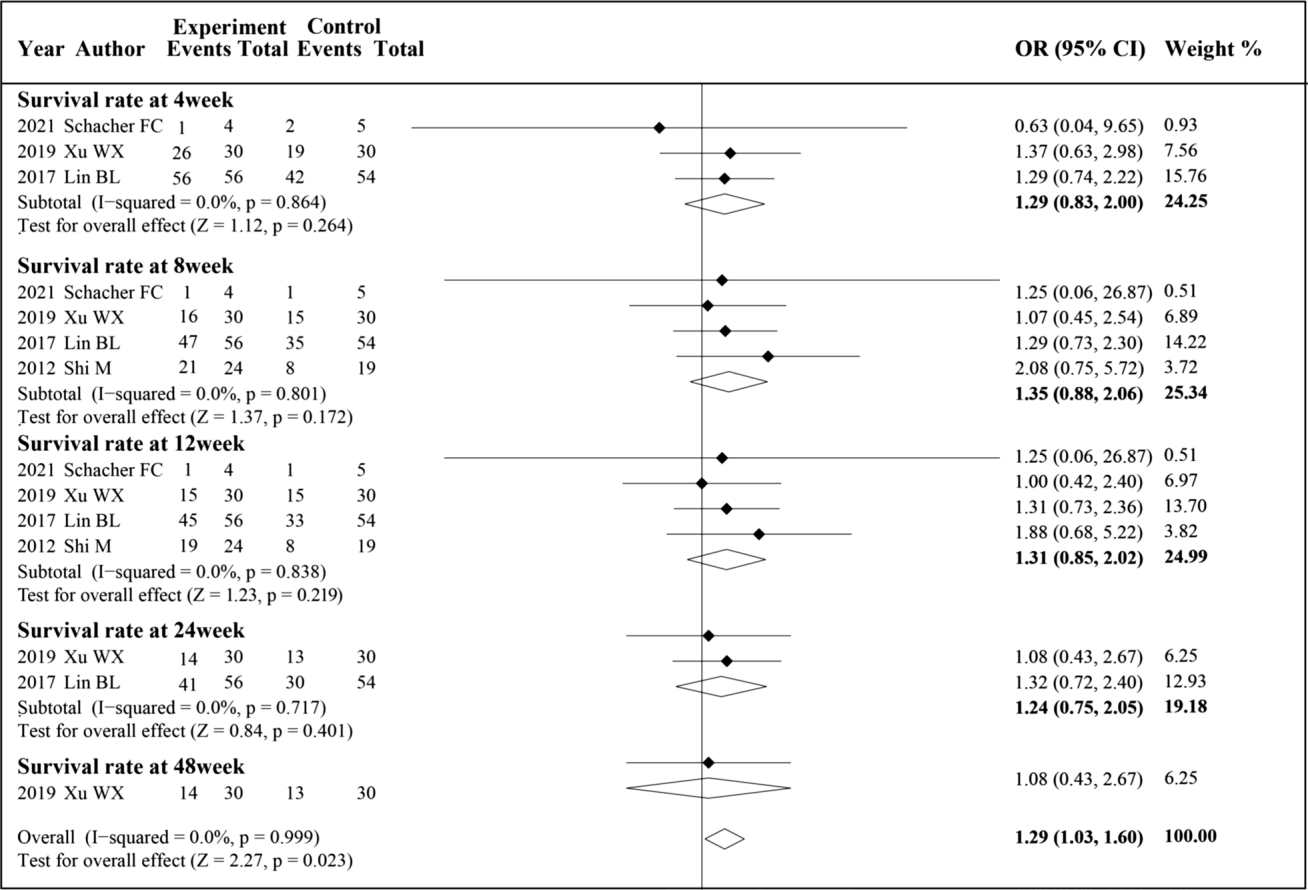
Forest plot of the comparison of the effect of MSCs therapy versus conventional treatment on survival rate

We analysis the survival rate of patients at 4 weeks, 8 weeks, 12 weeks, 24 weeks and 48 weeks. There were no significant statistical heterogeneities at any time point; therefore, the fixed effects model was used. Compared with the control group, MSCs therapy did not show significant differences at all the time points. However, it showed a trend of a higher survival rate at each stage of treatment, and the pooled OR indicated a significant increase in the survival rate in patients with MSCs therapy (OR 1.29, 95% CI 1.03–1.60; *P* = 0.023) (Fig. 3). Due to the insufficient number of included studies, we did not perform subgroup analysis.

### MELD score

**Fig. 4:**
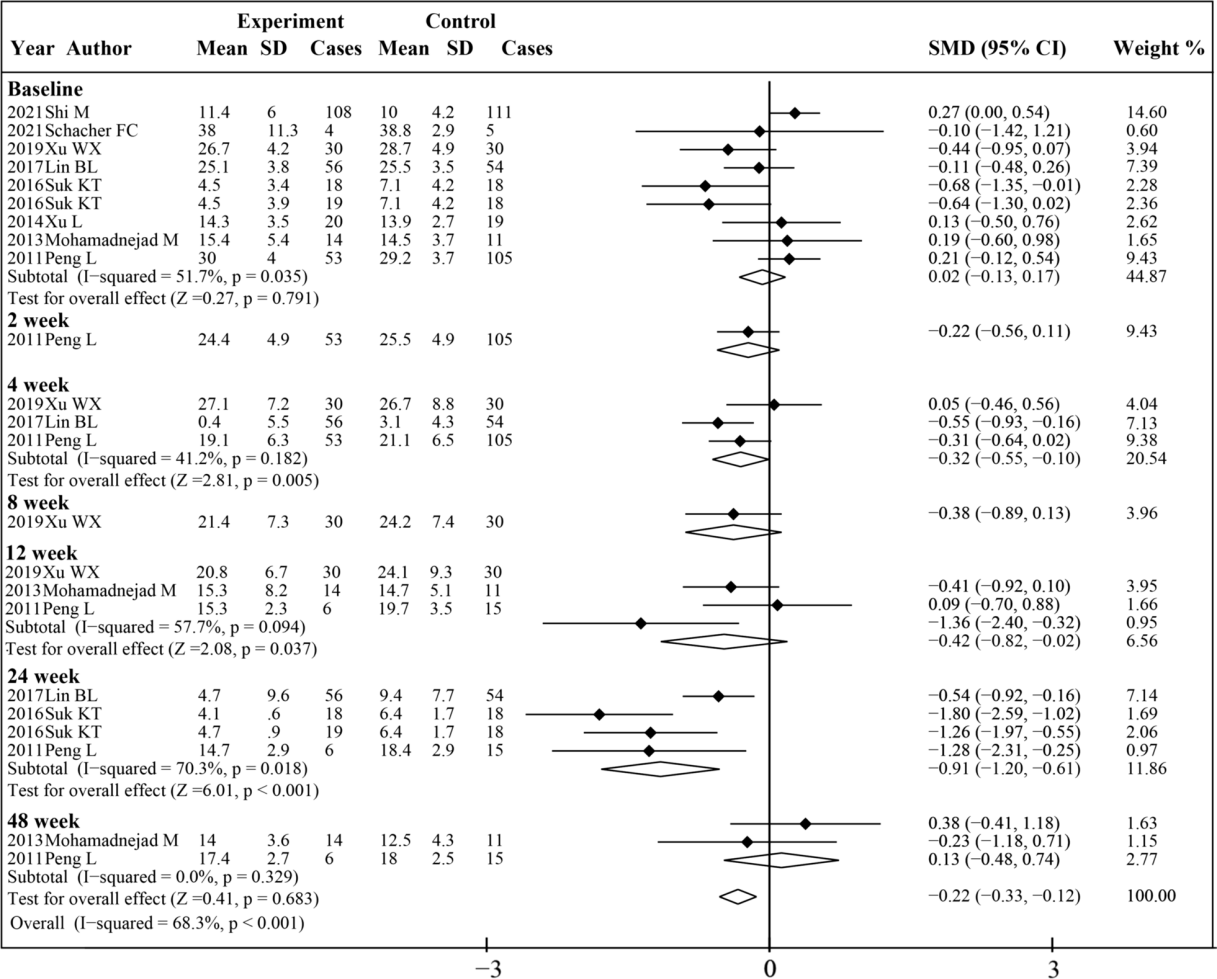
Forest plot of the comparison of the effect of MSCs therapy versus conventional treatment on MELD score

Eight studies were included in the analysis of the MELD score (Fig. 4). Before treatment, no significant difference was observed between the experiment group and control group (SMD 0.02, 95% CI  − 0.13 to 0.17; *P* = 0.791). The MELD score significantly decreased at 4 weeks (SMD − 0.32, 95% CI  − 0.55 to  − 0.10; *P* = 0.005), 12 weeks (SMD − 0.42, 95% CI − 0.82 to  − 0.02; *P* = 0.037) and 24 weeks (SMD − 0.091, 95% CI − 1.20 to  − 0.61; *P* < 0.001) after MSCs therapy. No significant difference was found compared to the control group after 48 weeks. Then, we conducted a subgroup analysis to explore the effects of MSCs therapy on MELD score by other factors such as different liver diseases background, administration routes, different cell types and times of treatment (Additional file 1: Table S1). MSCs therapy was associated with decreased MELD score at 4 weeks, 12 weeks and 24 weeks in the ACLF subgroup and at 24 weeks in the cirrhosis without ACLF subgroup. MELD score decreased significantly after MSCs therapy at 4 weeks in the intravenous injection subgroup and at 24 weeks in the hepatic arterial injection subgroup. MELD score decreased significantly after BM-MSCs therapy at 4 weeks and 24 weeks. In the UC-MSCs subgroup, comparison between the two groups could not be made due to a limited number of included studies. As for times of treatment, MSCs therapy was associated with decreased MELD score at 24 weeks in the single treatment subgroup and at 4 weeks and 24 weeks in the multiple treatment subgroup. As for etiology, MSCs therapy was associated with decreased MELD score at 4 weeks and 24 weeks in patients with liver disease caused by HBV.

We found substantial heterogeneity at 24 weeks (*I*^2^ = 70.3%). By excluding the results of Lin et al.[10], sensitivity analyses showed lowered heterogeneity among the remaining studies (Additional file 1: Table S2). Publication bias was evaluated at 24 weeks. The funnel plot, Egger’s test and Begg’s test indicated no evident publication bias (Additional file 2: Fig. S1).

### ALB level

**Fig. 5:**
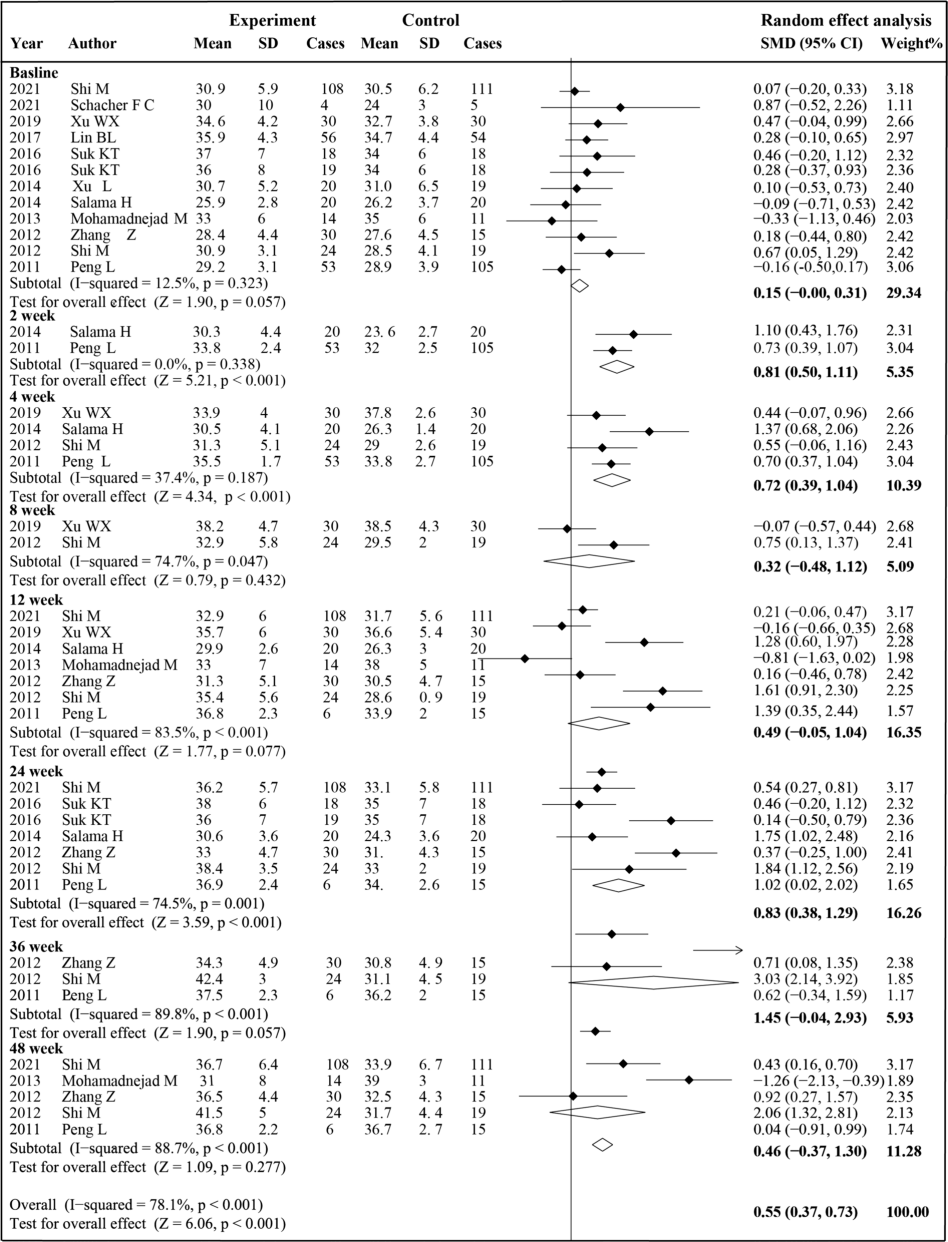
Forest plot of the comparison of the effect of MSCs therapy versus conventional treatment on albumin (ALB) level

Ten studies were included in the analysis of ALB levels (Fig. 5). Before treatment, no significant difference was observed between the two groups (SMD 0.15, 95% CI 0.00–0.31; *P* = 0.057). After MSCs treatment, the ALB levels increased significantly compared to the control group at 2 weeks (SMD 0.81, 95% CI 0.50–1.11; *P* < 0.001), 4 weeks (SMD 0.72; 95% CI 0.39–1.04; *P* < 0.001) and 24 weeks (SMD 0.83, 95% CI 0.38–1.29; *P* < 0.001).We also conduct a subgroup analysis to explore whether other factors influence ALB levels after different therapy (Additional file 1: Table S3). MSCs therapy was associated with increased ALB level at 4 weeks and 24 weeks in the ACLF subgroup, while only at 24 weeks in the cirrhosis without ACLF subgroup. No significant difference was observed between the MSCs group and the control group in the hepatic arterial injection subgroup. In the intravenous injection subgroup, the comparison between the two groups could not be made due to the difference in baseline results. MSCs therapy was associated with increased ALB levels at 2 weeks, 4 weeks and 24 weeks in the BM-MSCs subgroup and at 4 weeks, 24 weeks and 48 weeks in the UC-MSCs subgroup. MSCs therapy was associated with increased ALB levels at 2 weeks, 4 weeks and 24 weeks in the single treatment subgroup. In the multiple treatment subgroup, the comparison between the two groups could not be made due to the difference in baseline results. As for etiology, MSCs therapy was associated with increased ALB levels at 4 weeks, 24 weeks and 48 weeks in patients with liver disease caused by HBV.

We found substantial heterogeneity at most of the time points (*I*^2^ = 74–89%). However, sensitivity analyses did not show reduced heterogeneity (Additional file 1: Table S4). Publication bias was evaluated at 12, 24 and 48 weeks. The funnel plot, Egger’s test and Begg’s test indicated no evident publication bias (Additional file 2: Fig. S1).

### TBIL level

**Fig. 6:**
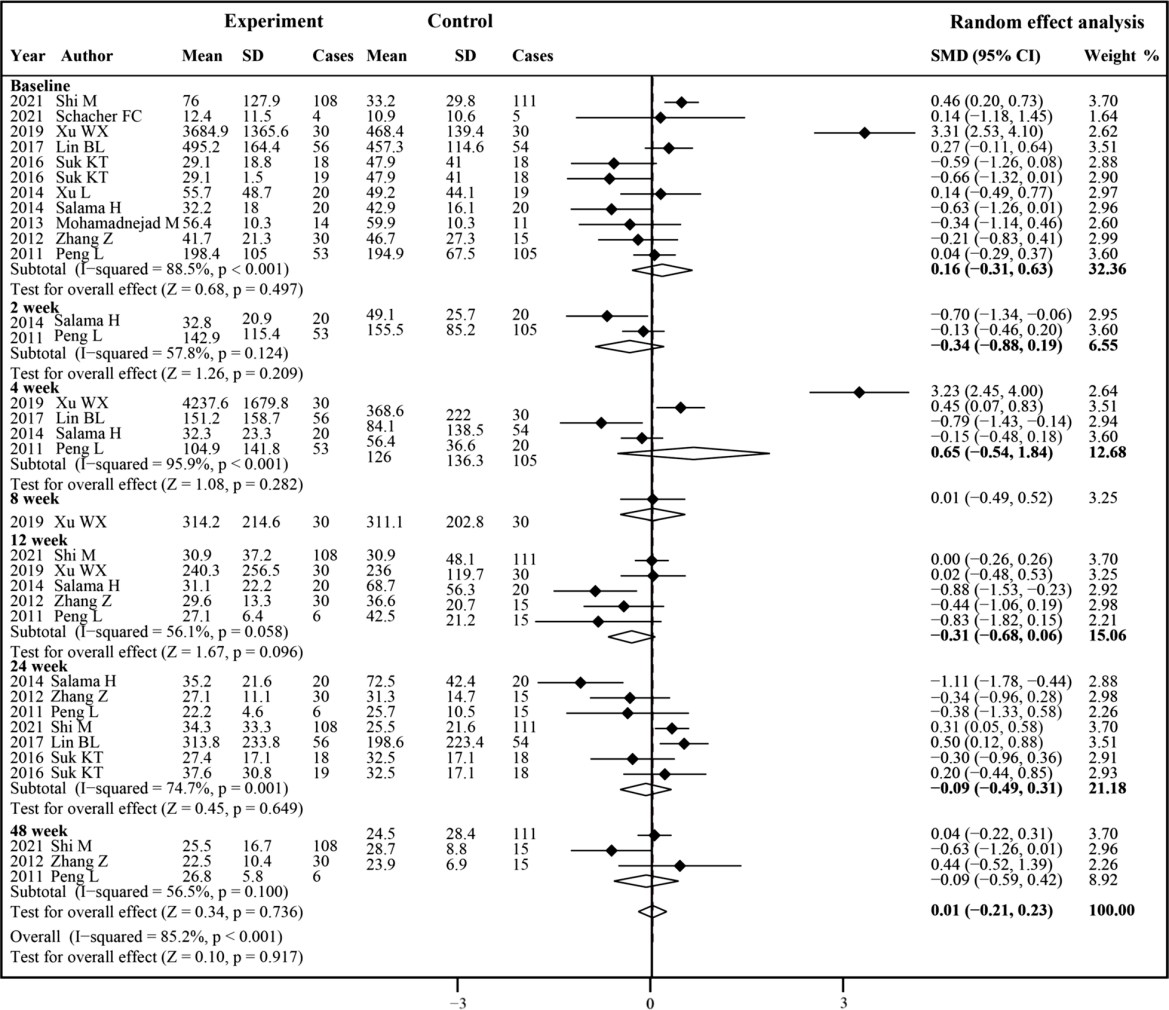
Forest plot of the comparison of the effect of MSCs therapy versus conventional treatment on total bilirubin (TBIL) level

Ten studies were included in the analysis of TBIL levels (Fig. 6). Before treatment, no significant difference was observed between the experiment group and control group (SMD 0.16, 95% CI − 0.31 to − 0.63; *P* = 0.497).However, no significant changes were found after both MSCs therapy and conventional treatment at all time points. Then, we conducted a subgroup analysis to explore whether the effects of MSCs therapy on TBIL levels by other factors (Additional file 1: Table S5). MSCs therapy was associated with decreased TBIL levels at 24 weeks in the BM-MSCs subgroup (SMD − 0.86, 95% CI − 1.41 to − 0.32; *P* = 0.002). TBIL levels also decreased after MSCs therapy compared with the control group in the single treatment subgroup at 12 weeks (SMD − 0.86, 95% CI − 1.41 to − 0.32; *P* = 0.002) and 24 weeks (SMD − 0.62, 95% CI − 1.17 to − 0.08; *P* = 0.025).

We found substantial heterogeneity at 4 weeks (*I*^2^ = 95.9%) and 24 weeks (*I*^2^ = 74.7%). By excluding the results of Salama et al.[9] at 24 weeks, sensitivity analyses showed lowered heterogeneity among the remaining studies (Additional file 1: Table S6). Publication bias was evaluated at 4 and 24 weeks. The funnel plot, Egger’s test and Begg’s test indicated no evident publication bias (Additional file 2: Fig. S1).

### Coagulation function (PTA and INR)

**Fig. 7:**
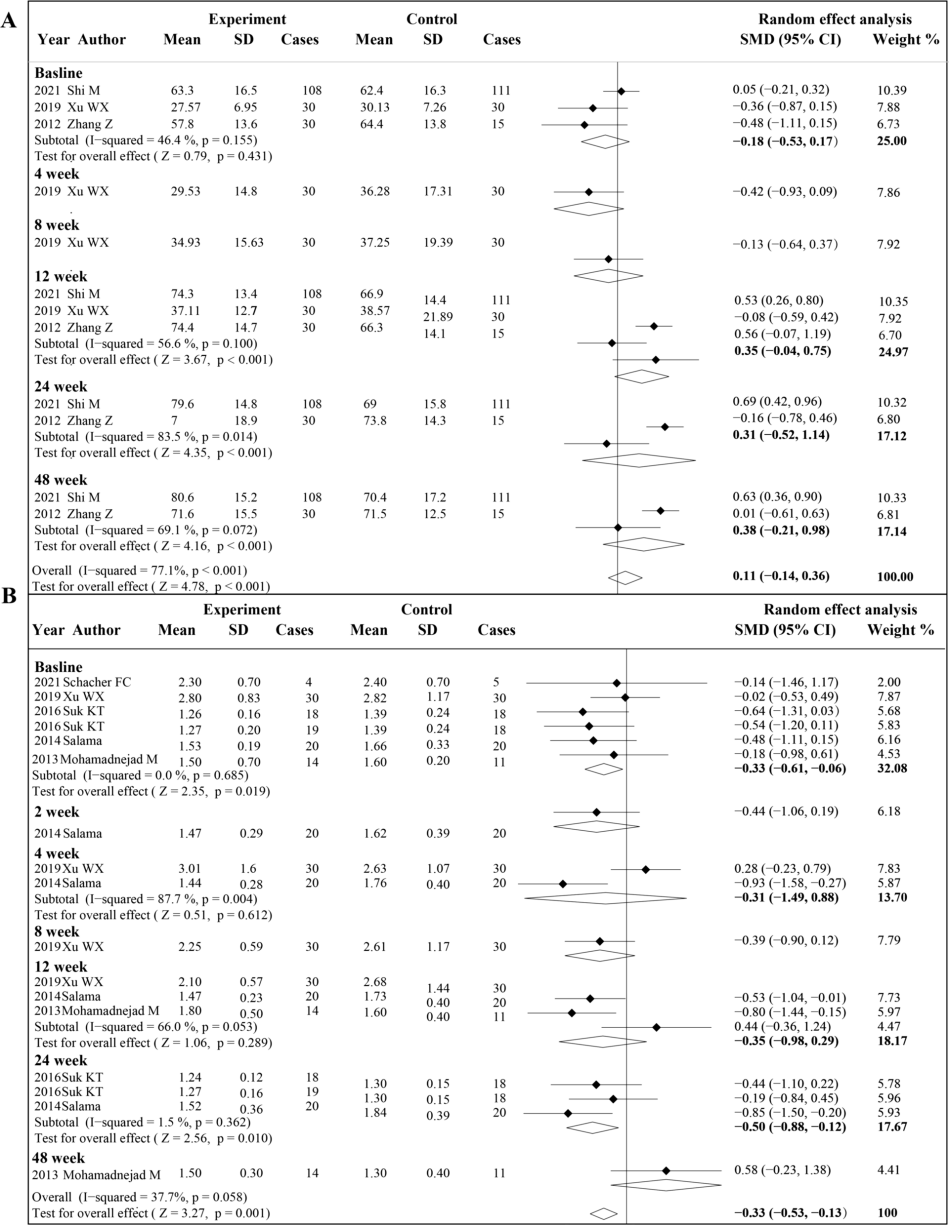
Forest plot of the comparison of the effect of MSCs therapy versus conventional treatment on coagulation function. **A** Forest plot of prothrombin activity (PTA), **B** forest plot of international normalized ratio (INR)

Three studies and five studies were included in the analysis of the PTA level and INR level, respectively (Fig. 7). Before treatment, no significant difference in PTA level was observed between the experiment group and control group (SMD − 0.18, 95% CI − 0.53 to − 0.17; *P* = 0.431). After MSCs treatment, the PTA level increased significantly compared to the control group at 12 weeks (SMD 0.35, 95% CI − 0.04 to 0.75; *P* < 0.001), 24 weeks (SMD 0.31; 95% CI − 0.52 to 1.14; *P* < 0.001) and 48 weeks (SMD 0.38, 95% CI − 0.14 to 0.36; *P* < 0.001). As for INR level, the comparison between the two groups could not be made due to the difference before treatment (SMD − 0.33, 95% CI − 0.61 to  − 0.06; *P* = 0.019). Due to the insufficient number of included studies, we did not perform subgroup analysis.

### Transaminase level (ALT and AST)

**Fig. 8:**
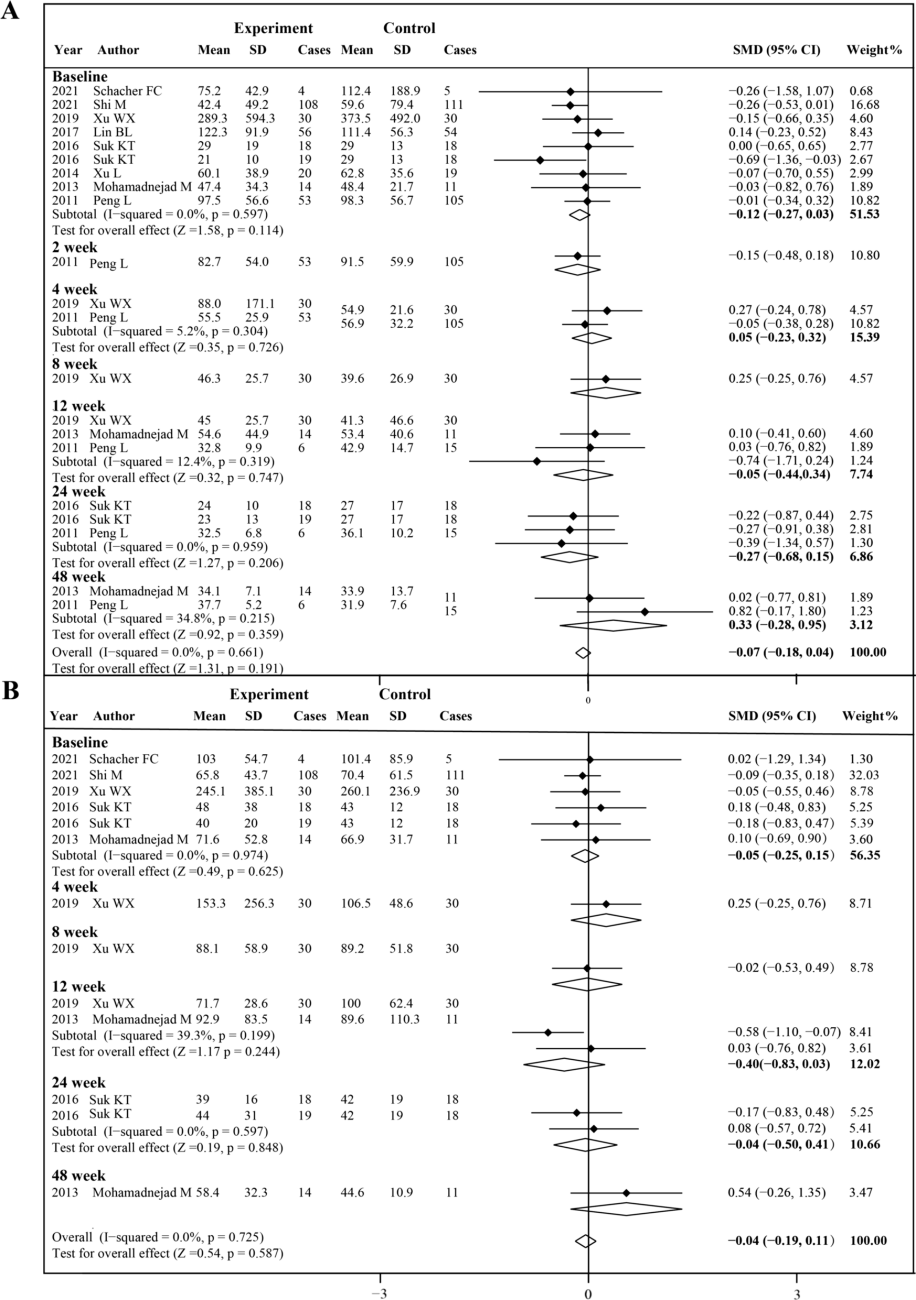
Forest plot of the comparison of the effect of MSCs therapy versus conventional treatment on transaminase. **A** Forest plot of alanine aminotransferase (ALT), **B** forest plot of aspartate aminotransferase (AST).

Eight studies and five studies were included in the analysis of ALT level and AST level, respectively (Fig. 8). There were no significant statistical heterogeneities at any time point; therefore, the fixed effects model was used. Before treatment, no significant difference was observed between the experiment group and control group of ALT level (SMD − 0.12, 95% CI − 0.27 to − 0.03; *P* = 0.114) and AST level (SMD − 0.05, 95% CI − 0.25 to − 0.15; *P* = 0.974). However, no significant changes were found after both MSCs therapy and conventional treatment at all time points. Due to the insufficient number of included studies, we did not perform subgroup analysis.

### Adverse events and clinical symptoms assessment

Seven studies reported that there were no statistically significant side effects or complications related to cell infusion, while five studies reported that the only adverse event of the MSCs therapy was fever. Analysis on clinical symptoms showed that encephalopathy and gastrointestinal hemorrhage events were significantly reduced in patients after MSCs therapy, while no significant difference was observed of rash, itching and edema rate between the MSCs therapy group and the control group (Table 2).

**Table 2:** Meta-analysis of clinical symptoms in pre- and post-therapy

Symptom	Time point	Number of studies	Heterogeneity		Odds ratio (OR)	95% CI	*P* value
			I^2^	*P* value			
Encephalopathy	Baseline	2	0	0.457	0.65	0.30–1.41	0.278
	End of treatment	2	31.8	0.231	0.40	0.18–0.89	0.025
Gastrointestinal hemorrhage	Baseline	3	0	0.491	0.80	0.19–3.35	0.760
	End of treatment	3	1.9	0.361	0.32	0.07–0.97	0.045
Edema	Baseline	4	0	0.379	1.33	0.58–3.06	0.495
	End of treatment	4	46.6	0.132	0.64	0.27–1.49	0.298
Rash	Baseline	4	0	0.491	1.11	0.22–5.73	0.900
	End of treatment	4	9.2	0.347	0.73	0.26–2.07	0.555
Itching	Baseline	2	0	0.491	0.44	0.06–3.16	0.417
	End of treatment	2	57.2	0.127	0.45	0.08–2.45	0.352

95% CI, 95% confidence interval

## Discussion

In recent years, stem cells therapy for different liver diseases has been thoroughly investigated. It is proven to improve liver function, reverse fibrosis, relieve clinical symptoms and reduce mortality in both animal models and clinical trials [28–31]. Several meta-analysis studies [32–34] also demonstrated that stem cell therapy is a safe and effective treatment for patients with chronic liver disease. MSCs are a kind of multipotent stem cells and have been considered the most promising cells for regeneration, transplantation and cell therapy. The main objective of our study was to assess the efficacy and safety of MSCs therapy in patients with chronic liver disease. Our results indicated that MSCs treatment could improve liver function which was mainly reflected by the level of ALB, TBIL, MELD score and coagulation function but did not alter the ALT and AST compared with the conventional treatment group. No significant change in survival rate was shown after MSCs therapy; however, there was a slightly positive trend, and a pooled OR indicated the increase in survival rate in patients with MSCs therapy.

ALB and coagulation factors are mainly synthesized in the liver. They can appropriately reflect the liver reserve function in patients with cirrhosis and liver failure. We demonstrated that the ALB levels and PTA increased after MSCs therapy. That indicated MSCs could contribute to liver regeneration. TBIL and transaminase are parameters to show the severity of the liver injury. Our results showed that the TBIL levels in the MSCs group decreased in the BM-MSCs subgroup. However, no significant difference in ALT and AST levels was found after MSCs therapy. In clinical practice, we found that ALT elevated earlier than TBIL in patients with end-stage liver disease. When hepatocytes are destroyed to some extent, ALT and AST levels start to decline, while TBIL levels persistently elevate. That may be one of the main reasons why no difference in ALT levels was seen between the two study groups. This also confirmed the results [10, 11, 21, 25] of several clinical studies and matched the opinion of Lin et al. [10]. In addition, the difference in disease types and disease etiology, as well as the limited number of patients, might be the cause of insignificance in the improvements in ALT and AST in our study. The MELD score [35] is designed to predict survival in end-stage liver disease using serum bilirubin, INR and creatinine. Our study showed a decreased MELD score after MSCs therapy, which was consistent with previous clinical trials [8, 23, 36]. However, it should be noted that there was no difference in MELD score at 48 weeks and even an increasing trend after MSCs therapy. The limited number of included studies could be one of the reasons. In addition, the change of MELD scores is related to natural courses and laboratory parameters fluctuations and probably does not reflect the change in mortality rate [23, 37]. In our study, no significant difference in survival rate was shown after MSCs therapy at all time points. However, we only compared short-term survival rate within 48 weeks. Shi et al. [27] conducted an RCT study recently and discovered no difference of survival rate between the UC-MSCs group and the control group within 13 months, but the survival rate was higher after UC-MSCs treatment during the 13–75-month follow-up. MSCs therapy might exert a better effect on the long-term survival rate.

As safety is a major concern in the clinical application of MSCs therapy, our analysis evaluated the safety and the change in clinical symptoms of MSCs therapy for treating chronic liver disease. Fever was the only side effect. No serious adverse events or death related to the MSCs treatment was reported. We also found that MSCs therapy significantly reduced the risk of encephalopathy and gastrointestinal hemorrhage, while there was no difference in edema, rash and itching between the two groups. Nevertheless, some clinical studies suggested that MSCs therapy had security risks including immune reactivity, tumorigenic potential and even death [38, 39]. Liang et al. [15] treated six patients with autoimmune liver disease through peripheral intravenous infusion of BM-MSCs. The results showed that not only did clinical symptoms of the patients worsen, but two deaths occurred. Further high-quality clinical studies with a larger sample size and longer follow-up period are still in demand to evaluate the safety of MSCs therapy.

To explore whether other factors influence the therapeutic efficacy of cell transplantation, we performed subgroup analyses according to liver disease population, cell type, delivery route and injection frequency. Most clinical researches on liver diseases including ACLF and cirrhosis showed that patients could benefit from MSCs treatment. Our subgroup analyses indicate that patients with ACLF and cirrhosis without ACLF both had improved liver function with increased ALB levels and decreased MELD score. Liver transplantation is considered the only curative treatment for end-stage liver disease and ACLF at present. Thus, MSCs therapy could develop a potential alternative to liver transplantation. We also compared different kinds of MSCs regarding efficacy for chronic liver disease. Our study showed that BM-MSCs and UC-MSCs treatment had similar efficacy to improve liver function. That was not consistent with the analysis results of Zhou et al. [32] that suggested that BM-MSCs had superior therapeutic effects to UC-MSCs. UC-MSCs are ideal MSC resources for their relative ease of collection, low alloreactivity and young cellular age. Therefore, more clinical trials should be conducted to compare the therapeutic effects between BM-MSCs and UC-MSCs treatment.

Across the included studies, MSCs were transplanted into the liver either through the peripheral vein or through the hepatic artery. Peripheral intravenous infusion has been considered an ideal administration route as it is easy and convenient to perform and MSCs migrate well into liver parenchyma differentiate into hepatocytes in the context of chronic injury in vivo [39]. Whether hepatic arterial injection is feasible has been controversial. For one thing, it is an invasive procedure with potential risks of portal hypertensive bleeding and thrombosis. However, some studies suggested that hepatic arterial injection was more effective than the peripheral vein because of less loss and higher homing ability of MSCs during the treatment [32, 40]. Our study suggested that the effectiveness varied slightly between the two administration routes. Nonetheless, as a systemic administration, whatever the delivery route, side effects of therapy such as immune reaction and bleeding should be closely observed. There is no consensus on the times of MSCs treatment for chronic liver disease. Our results showed that multiple injections exerted greater benefit on the MELD score, while a single administration had more favorable effects on ALB levels. More clinical studies should be conducted to determine the optimal time of treatments.

This study had some limitations. Firstly, there was significant between-study heterogeneity. Although we used random-effects model and performed sensitivity analyses, heterogeneity could not be eliminated. And it was impossible to conduct all of the subgroup analyses to find the source of heterogeneity due to the limited including studies. Secondly, we compared the data on the premise of no difference in the baseline. Because the included studies were not consistent at different time points, it was difficult to summarize robust results at a specific time point. Moreover, on the premise that the baseline was different, it was inappropriate to compare the two groups. Thirdly, most of the included studies lack large size patients and long-term follow-up period which prevent definite conclusions from being made about the safety and efficacy of MSCs therapy in liver diseases.

## Conclusion

Our study suggested that MSCs therapy can improve liver function and alleviate clinical symptoms without serious adverse events. It had therapeutic effects on patients with both ACLF and cirrhosis. BM-MSCs and UC-MSCs treatment had similar efficacy to improve liver function. The effectiveness varied slightly between the peripheral intravenous injection and hepatic arterial injection. However, many concerns including the optimization of cell source, cell dosage, injection frequency and administration route must be addressed before clinical routine applications. Therefore, the protocol for MSCs therapy in different chronic liver diseases should be further refined, and its efficacy and safety should be further assessed in randomized trials with a large cohort study.

## Supplementary Information

**Additional file 1.** **Table S1.** Results of subgroup analyses of the effect of MSCs therapy on MELD score. **Table S2.** Results of sensitivity analyses of the effect of MSCs therapy on MELD score. **Table S3.** Results of subgroup analyses of the effect of MSCs therapy on ALB level. **Table S4.** Results of sensitivity analyses of the effect of MSCs therapy on ALB level. **Table S5.** Results of subgroup analyses of the effect of MSCs therapy on TBIL level. **Table S6.** Results of sensitivity analyses of the effect of MSCs therapy on TBIL level.

**Additional file 2: Figure S1.** Funnel plots of MELD score at 24 weeks; ALB levels at 12 weeks, 24 weeks, and 48 weeks; TBIL at 4 weeks and 24 weeks; No asymmetry was observed in the funnel plots. No publication bias was found using Egger’s and Begg’s test.

## Data Availability

The data used to support the findings of this study are available from the corresponding author upon request.

## Abbreviations

MSCs: Mesenchymal stem cells
RCTs: Randomized controlled trials
MELD: Model of end-stage liver disease
ACLF: Acute on chronic liver failure
BM-MSCs: Bone marrow-derived MSCs
UC-MSCs: Umbilical cord-derived MSCs
ALB: Albumin
TBIL: Total bilirubin
PTA: Prothrombin activity
INR: International normalized ratio
ALT: Alanine aminotransferase
AST: Aspartate aminotransferase
SMD: Standardized mean difference
OR: Odds ratio
95% CI: 95% Confidence interval
