# Supplementary material for: The assessment of mesenchymal stem cells therapy in acute on chronic liver failure and chronic liver disease: a systematic review and meta-analysis of randomized controlled clinical trials
Source: Stem Cell Res Ther. 2022 May 16;13:204. doi: 10.1186/s13287-022-02882-4 (PMC9109309; doi:10.1186/s13287-022-02882-4)
Supplement: Supplementary file 1 — Additional file 1. Table S1. Results of subgroup analyses of the effect of MSCs therapy on MELD score. Table S2. Results of sensitivity analyses of the effect of MSCs therapy on MELD score. Table S3. Results of subgroup analyses of the effect of MSCs therapy on ALB level. Table S4. Results of sensitivity analyses of the effect of MSCs therapy on ALB level. Table S5. Results of subgroup analyses of the effect of MSCs therapy on TBIL level. Table S6. Results of sensitivity analyses of the effect of MSCs therapy on TBIL level. [file 13287_2022_2882_MOESM1_ESM.docx]

**Additional file 1: Table S1.** Results of subgroup analyses of the effect of MSCs therapy on MELD score.

|  | **Time point** | **Heterogeneity** | | **SMD** | **95%-CI** | ***Z*** | ***p*-value** |
| --- | --- | --- | --- | --- | --- | --- | --- |
| **Liver diesease** |  | ***I2*** | ***p-value*** |  |  |  |  |
| ACLF | Baseline | 35.5% | 0.199 | -0.03 | [-0.25, 0.19] | 0.26 | 0.798 |
|  | **4 week** | **41.2%** | **0.182** | **-0.32** | **[-0.55, -0.10]** | **2.81** | **0.005** |
|  | **12 week** | **61.7%** | **0.106** | **-0.59** | **[-1.05, -0.13]** | **2.53** | **0.011** |
|  | **24 week** | **42.2%** | **0.188** | **-0.63** | **[-0.98, -0.27]** | **3.44** | **0.001** |
|  | **Overall** | **44.0%** | **0.039** | **-0.28** | **[-0.40, 0.15]** | **4.42** | **0.000** |
| Cirrhosis without ACLF | Baseline | 65.3% | 0.021 | 0.07 | [-0.14, 0.28] | 0.61 | 0.542 |
|  | **24 week** | **2.1%** | **0.312** | **-1.51** | **[-2.03, -0.98]** | **5.62** | **0.000** |
|  | Overall | 81.9% | 0.000 | -0.11 | [-0.29, 0.07] | 0.73 | 0.243 |
| **Administration route** |  |  |  |  |  |  |  |
| Intravenous injection | Baseline | 42.8% | 0.136 | 0.06 | [-0.13, 0.25] | 0.61 | 0.544 |
|  | **4 week** | **70.5%** | **0.065** | **-0.33** | **[-0.63, -0.33]** | **2.13** | **0.033** |
|  | 12 week | 5.0% | 0.305 | -0.26 | [-0.69, 0.17] | 1.19 | 0.232 |
|  | **Overall** | **54.4%** | **0.012** | **-0.14** | **[-0.28, -0.01]** | **2.09** | **0.037** |
| Hepatic arterial injection | Baseline | 67.1% | 0.028 | -0.04 | [-0.29,0.20] | 0.35 | 0.723 |
|  | **24 week** | **0.0%** | **0.556** | **-1.46** | **[-1.93,-0.99]** | **6.11** | **0.000** |
|  | **Overall** | **76.1%** | **0.000** | **-0.34** | **[-0.49,-0.18]** | **4.22** | **0.000** |
| **Cell type** |  |  |  |  |  |  |  |
| BM-MSC | Baseline | 37.4% | 0.143 | -0.09 | [-0.33, 0.15] | 0.49 | 0.623 |
|  | **4 week** | **0.0%** | **0.362** | **0.49** | **[-0.37, 1.36]** | **3.23** | **0.001** |
|  | 12 week | 78.8% | 0.030 | 0.55 | [-1.38, 1.48] | 1.38 | 0.167 |
|  | **24 week** | **70.3%** | **0.018** | **-0.39** | **[-1.63, 0.86]** | **6.01** | **0.000** |
|  | 48 week | 0.0% | 0.329 | 0.13 | [-0.48,0.74] | 0.41 | 0.683 |
|  | **Overall** | **0.670** | **0.000** | **0.01** | **[-0.29, 0.31]** | **5.03** | **0.000** |
| **Times of treatment** |  |  |  |  |  |  |  |
| Single | Baseline | 31.3% | 0.214 | 0.00 | [-0.31, 0.31] | 0.52 | 0.603 |
|  | 12 week | 78.8% | 0.030 | -0.44 | [-1.07, 0.19] | 1.38 | 0.167 |
|  | **24 week** | **0.0%** | **0.423** | **-1.61** | **[-2.23, -0.99]** | **5.07** | **0.000** |
|  | 48 week | 0.0% | 0.329 | 0.13 | [-0.48,0.74] | 0.41 | 0.683 |
|  | **Overall** | **71.0%** | **0.000** | **-0.21** | **[-0.37, -0.06]** | **2.67** | **0.008** |
| Multiple | Baseline | 62.8% | 0.029 | -0.15 | [-0.52,0.21] | 0.06 | 0.955 |
|  | **4 week** | **70.5%** | **0.065** | **-0.33** | **[-0.63,-0.03]** | **2.13** | **0.033** |
|  | **24 week** | **67.6%** | **0.079** | **-0.70** | **[-1.04,-0.36]** | **4.09** | **0.000** |
|  | **Overall** | **68.2%** | **0.001** | **-0.23** | **[-0.37,-0.10]** | **3.42** | **0.001** |
| **Etiology** |  |  |  |  |  |  |  |
| HBV | Baseline | 46.4% | 0.113 | 0.06 | [-0.18, 0.29] | 0.45 | 0.652 |
|  | **4 week** | **41.2%** | **0.182** | **-0.30** | **[-0.60, -0.01]** | **1.99** | **0.046** |
|  | 12 week | 61.7% | 0.106 | -0.77 | [-1.68, 0.14] | 1.66 | 0.096 |
|  | **24 week** | **42.2%** | **0.188** | **-0.75** | **[-1.39, -0.10]** | **2.25** | **0.024** |
|  | **Overall** | **62.6%** | **0.001** | **-0.25** | **[-0.44, -0.05]** | **2.51** | **0.012** |

**Additional file 1: Table S2.** Results of sensitivity analyses of the effect of MSCs therapy on MELD score.

|  | **Random-effects model (Inverse-Variance)** | | | | |
| --- | --- | --- | --- | --- | --- |
| **MELD at 24 week** | **SMD 95%-CI** | ***Z*** | **p-value** | ***I2*** | ***p*** |
| Pooled estimate | -1.16 [-1.78, -0.53] | 3.60 | 0.000 | 70.3% | 0.018 |
| Omitting 2011 Peng L | -1.14 [-1.92, -0.37] | 2.89 | 0.004 | 79.1% | 0.008 |
| Omitting 2016 Suk KT | -1.15 [-2.01, -0.28] | 2.60 | 0.009 | 76.6% | 0.011 |
| Omitting 2016 Suk KT | -0.91 [-1.46, -0.36] | 4.53 | 0.000 | 52.0% | 0.124 |
| Omitting 2017 Lin BL | -1.46 [-1.93, -0.99] | 6.11 | 0.000 | 0.0% | 0.566 |

**Additional file 1: Table S3.** Results of subgroup analyses of the effect of MSCs therapy on ALB level.

|  | **Time point** | **Heterogeneity** | | **SMD** | **95%-CI** | ***Z*** | ***p*-value** |
| --- | --- | --- | --- | --- | --- | --- | --- |
| **Liver diesease** |  | ***I2*** | ***p-value*** |  |  |  |  |
| ACLF | Baseline | 56.6% | 0.061 | 0.29 | [-0.06, 0.64] | 1.64 | 0.100 |
|  | **4 week** | **0.0%** | **0.692** | **0.61** | **[0.35, 0.87]** | **4.67** | **0.000** |
|  | 8 week | 74.7% | 0.047 | 0.32 | [-0.48, 1.12] | 0.79 | 0.432 |
|  | 12 week | 89.4% | 0.000 | 0.91 | [-0.36, 2.18] | 1.40 | 0.161 |
|  | **24 week** | **40.8%** | **0.194** | **1.51** | **[0.72, 2.29]** | **3.75** | **0.000** |
|  | 36 week | 92.2% | 0.000 | 1.83 | [-0.52, 4.19] | 1.53 | 0.127 |
|  | 48 week | 90.8% | 0.001 | 1.07 | [-0.91, 3.06] | 1.06 | 0.289 |
|  | **Overall** | **82.2%** | **0.000** | **0.77** | **[0.47, 1.08]** | **4.97** | **0.000** |
| Cirrhosis without ACLF | Baseline | 0.0% | 0.801 | 0.09 | [-0.10, 0.28] | 0.97 | 0.333 |
|  | 12 week | 80.3% | 0.002 | 0.24 | [-0.40,0.87] | 0.73 | 0.465 |
|  | **24 week** | **67.1%** | **0.016** | **0.62** | **[0.19, 1.05]** | **2.81** | **0.005** |
|  | 48 week | 87.8% | 0.000 | 0.09 | [-0.86, 1.04] | 0.19 | 0.850 |
|  | **Overall** | **71.5%** | **0.000** | **0.38** | **[0.17, 0.59]** | **3.55** | **0.000** |
| **Administration route** |  |  |  |  |  |  |  |
| Intravenous injection | **Baseline** | **11.5%** | **0.341** | **0.20** | **[0.01, 0.39]** | **2.07** | **0.039** |
|  | 4 week | 58.3% | 0.091 | 0.75 | [0.21, 1.29] | 2.74 | 0.006 |
|  | 8 week | 74.7% | 0.047 | 0.32 | [-0.48, 1.12] | 0.79 | 0.432 |
|  | 12 week | 84.5% | 0.000 | 0.38 | [-0.19, 0.95] | 1.31 | 0.189 |
|  | 24 week | 84.7% | 0.000 | 1.08 | [0.37, 1.79] | 2.97 | 0.003 |
|  | 48 week | 91.3% | 0.000 | 0.56 | [-0.44, 1.55] | 1.09 | 0.275 |
|  | Overall | 82.1% | 0.000 | 0.60 | [0.37, 0.83] | 5.15 | 0.000 |
| Hepatic arterial injection | Baseline | 0.0% | 0.801 | 0.09 | [-0.10,0.28] | 0.42 | 0.673 |
|  | 24 week | 4.9% | 0.349 | 0.43 | [0.00,0.86] | 1.94 | 0.052 |
|  | **Overall** | **56.4%** | **0.008** | **0.43** | **[0.17,0.69]** | **3.25** | **0.001** |
| **Cell type** |  |  |  |  |  |  |  |
| BM-MSC | Baseline | 4.6% | 0.395 | 0.08 | [-0.12, 0.28] | 0.79 | 0.428 |
|  | **2 week** | **0.0%** | **0.338** | **0.81** | **[0.50, 1.11]** | **5.21** | **0.000** |
|  | **4 week** | **65.2%** | **0.090** | **0.97** | **[0.33, 1.60]** | **2.97** | **0.003** |
|  | 12 week | 88.4% | 0.000 | 0.61 | [-0.81, 2.04] | 0.85 | 0.397 |
|  | **24 week** | **74.0%** | **0.009** | **0.82** | **[0.09, 1.55]** | **2.20** | **0.028** |
|  | 48 week | 74.6% | 0.047 | -0.63 | [-1.90,0.65] | 0.96 | 0.336 |
|  | **Overall** | **75.3%** | **0.000** | **0.44** | **[0.17, 0.71]** | **3.22** | **0.001** |
| UC-MSC | Baseline | 30.1% | 0.231 | 0.27 | [-0.01, 0.55] | 1.90 | 0.057 |
|  | **4 week** | **0.0%** | **0.000** | **0.49** | **[0.09,0.88]** | **2.43** | **0.015** |
|  | 8 week | 74.7% | 0.047 | 0.32 | [-0.48,1.12] | 0.79 | 0.432 |
|  | 12 week | 82.6% | 0.001 | 0.41 | [-0.18,1.00] | 1.36 | 0.175 |
|  | **24 week** | **83.1%** | **0.003** | **0.87** | **[0.13,1.61]** | **2.30** | **0.022** |
|  | 36 week | 94.2% | 0.000 | 1.85 | [-0.42,4.11] | 1.60 | 0.110 |
|  | **48 week** | **88.2%** | **0.000** | **1.09** | **[0.17,2.00]** | **2.32** | **0.020** |
|  | **Overall** | **81.3%** | **0.000** | **-0.14** | **[-0.48,0.19]** | **5.20** | **0.000** |
| **Times of treatment** |  |  |  |  |  |  |  |
| Single | Baseline | 0.0% | 0.497 | -0.01 | [-0.23, 0.21] | 0.12 | 0.901 |
|  | **2 week** | **0.0%** | **0.338** | **0.81** | **[0.50,1.11]** | **5.21** | **0.000** |
|  | **4 week** | **65.2%** | **0.090** | **0.97** | **[0.33, 1.60]** | **2.97** | **0.003** |
|  | 12 week | 88.4% | 0.000 | 0.61 | [-0.81, 2.04] | 0.85 | 0.397 |
|  | **24 week** | **74.0%** | **0.009** | **0.82** | **[0.09, 1.55]** | **2.20** | **0.028** |
|  | 48 week | 74.6% | 0.047 | -0.63 | [-1.90,0.65] | 0.96 | 0.336 |
|  | **Overall** | **77.4%** | **0.000** | **0.44** | **[0.14, 0.73]** | **2.92** | **0.003** |
| Multiple | **Baseline** | **3.3%** | **0.395** | **0.25** | **[0.06,0.43]** | **2.58** | **0.010** |
|  | 4 week | 0.0% | 0.798 | 0.49 | [0.09,0.88] | 2.43 | 0.015 |
|  | 8 week | 74.7% | 0.047 | 0.32 | [-0.48,1.12] | 0.79 | 0.432 |
|  | 12 week | 82.6% | 0.001 | 0.41 | [-0.18,1.00] | 1.36 | 0.175 |
|  | 24 week | 83.1% | 0.003 | 0.87 | [0.13,1.61] | 2.30 | 0.022 |
|  | 36 week | 94.2% | 0.000 | 1.85 | [-0.42,4.11] | 1.60 | 0.110 |
|  | 48 week | 88.2% | 0.000 | 1.09 | [0.17,2.00] | 2.32 | 0.020 |
|  | Overall | 79.6% | 0.000 | 0.64 | [0.41,0.87] | 5.42 | 0.000 |
| **Etiology** |  |  |  |  |  |  |  |
| HBV | Baseline | 29.7% | 0.201 | 0.16 | [-0.03, 0.36] | 1.65 | 0.100 |
|  | **4 week** | **0.0%** | **0.692** | **0.61** | **[0.35, 0.87]** | **4.67** | **0.000** |
|  | 8 week | 74.7% | 0.047 | 0.32 | [-0.48, 1.12] | 0.79 | 0.432 |
|  | 12 week | 81.4% | 0.009 | 0.55 | [-0.02, 1.12] | 1.88 | 0.060 |
|  | **24 week** | **75.7%** | **0.006** | **0.89** | **[0.28, 1.50]** | **2.86** | **0.004** |
|  | 36 week | 89.8% | 0.000 | 1.45 | [-0.04, 2.93] | 1.90 | 0.057 |
|  | **48 week** | **83.8%** | **0.000** | **0.86** | **[0.11,1.61]** | **2.24** | **0.025** |
|  | **Overall** | **78.0%** | **0.000** | **0.60** | **[0.40, 0.79]** | **5.99** | **0.000** |

**Additional file 1: Table S4.** Results of sensitivity analyses of the effect of MSCs therapy on ALB level.

|  | **Random-effects model (Inverse-Variance)** | | | | |
| --- | --- | --- | --- | --- | --- |
| **ALB at 12 week** | **SMD 95%-CI** | ***Z*** | **p-value** | ***I2*** | ***p*** |
| Pooled estimate | 0.49[-0.05,1.04] | 1.77 | 0.077 | 83.5% | 0.000 |
| Omitting 2011 Peng L | 0.38[-0.19,0.95] | 1.31 | 0.189 | 84.5% | 0.000 |
| Omitting 2012 Shi M | 0.30[-0.20,0.80] | 1.16 | 0.244 | 77.5% | 0.000 |
| Omitting 2012 Zhang Z | 0.55[-0.09,1.20] | 1.69 | 0.091 | 86.1% | 0.000 |
| Omitting 2013 Mohamadnejad M | 0.67[0.12,1.23] | 2.40 | 0.016 | 82.6% | 0.000 |
| Omitting 2014 Salama H | 0.36[-0.21,0.92] | 1.24 | 0.213 | 82.8% | 0.000 |
| Omitting 2019 Xu WX | 0.62[-0.02,1.25] | 1.89 | 0.059 | 84.5% | 0.000 |
| Omitting 2021 Shi M | 0.56[-0.19,1.31] | 1.47 | 0.143 | 85.6% | 0.000 |
| **ALB at 24 week** | **SMD 95%-CI** | ***Z*** | **p-value** | ***I2*** | ***p*** |
| Pooled estimate | 0.83 [0.38, 1.29] | 3.59 | 0.000 | 74.5% | 0.001 |
| Omitting 2011 Peng L | 0.81 [0.31, 1.31] | 3.19 | 0.001 | 78.4% | 0.000 |
| Omitting 2012 Shi M | 0.66 [0.26, 1.05] | 3.27 | 0.001 | 61.2% | 0.024 |
| Omitting 2012 Zhang Z | 0.92[0.39,1.45] | 3.40 | 0.001 | 77.8% | 0.000 |
| Omitting 2014 Salama H | 0.68[0.26,1.10] | 3.17 | 0.002 | 66.2% | 0.011 |
| Omitting 2016 Suk KT | 0.95 [0.45, 1.64] | 3.70 | 0.000 | 75.8% | 0.001 |
| Omitting 2016 Suk KT | 0.90[0.37,1.43] | 3.33 | 0.001 | 78.4% | 0.000 |
| Omitting 2021 Shi M | 0.91 [0.31,1.51] | 2.96 | 0.003 | 76.5% | 0.000 |
| **ALB at 48 week** | **SMD 95%-CI** | ***Z*** | **p-value** | ***I2*** | ***p*** |
| Pooled estimate | 0.46 [-0.37, 1.30] | 1.09 | 0.277 | 88.7% | 0.000 |
| Omitting 2011 Peng L | 0.56 [-0.44, 1.55] | 1.09 | 0.275 | 91.3% | 0.000 |
| Omitting 2012 Shi M | 0.09 [-0.65, 0.84] | 0.24 | 0.807 | 93.1% | 0.001 |
| Omitting 2012 Zhang Z | 0.34 [-76, 1.44] | 0.61 | 0.545 | 91.0% | 0.000 |
| Omitting 2013 Mohamadnejad M | 0.86 [0.11, 1.61] | 2.24 | 0.025 | 83.8% | 0.000 |
| Omitting 2021 Shi M | 0.46[-0.89, 1.80] | 0.67 | 0.505 | 91.3% | 0.000 |

**Additional file 1: Table S5.** Results of subgroup analyses of the effect of MSCs therapy on TBIL level.

|  | **Time point** | **Heterogeneity** | | **SMD** | **95%-CI** | ***Z*** | ***p*-value** |
| --- | --- | --- | --- | --- | --- | --- | --- |
| **Liver diesease** |  | ***I2*** | ***p-value*** |  |  |  |  |
| ACLF | Baseline | 94.8% | 0.000 | 0.93 | [-0.24, 2.11] | 1.56 | 0.120 |
|  | 4 week | 96.8% | 0.000 | 1.12 | [-0.31, 2.56] | 1.54 | 0.124 |
|  | 12 week | 56.4% | 0.130 | -0.30 | [-1.11, 0.51] | 0.72 | 0.472 |
|  | 24 week | 64.5% | 0.093 | 0.18 | [-0.66, 1.01] | 0.42 | 0.676 |
|  | Overall | 90.6% | 0.000 | 0.48 | [0.04, 0.91] | 2.16 | 0.031 |
| Cirrhosis without ACLF | Baseline | 74.0% | 0.001 | -0.22 | [-0.64, 0.20] | 1.02 | 0.308 |
|  | 12 week | 70.7% | 0.033 | -0.37 | [-0.91, 0.17] | 1.36 | 0.174 |
|  | 24 week | 77.9% | 0.001 | -0.21 | [-0.72, 0.31] | 0.79 | 0.428 |
|  | 48 week | 72.5% | 0.056 | -0.23 | [-0.87, 0.42] | 0.69 | 0.489 |
|  | Overall | 71.5% | 0.000 | -0.28 | [-0.49, -0.07] | 2.56 | 0.011 |
| **Administration route** |  |  |  |  |  |  |  |
| Intravenous injection | Baseline | 91.5% | 0.000 | 0.42 | [-0.29, 1.14] | 1.15 | 0.248 |
|  | 4 week | 96.8% | 0.000 | 0.95 | [-0.94, 2.84] | 0.98 | 0.326 |
|  | 12 week | 58.7% | 0.064 | -0.25 | [-0.63, 0.13] | 1.29 | 0.196 |
|  | 24 week | 85.5% | 0.000 | -0.09 | [-0.68, 0.49] | 0.31 | 0.757 |
|  | 48 week | 72.5% | 0.056 | -0.23 | [-0.87, 0.42] | 0.69 | 0.489 |
|  | Overall | 89.3% | 0.000 | 0.11 | [-0.20, 0.41] | 0.70 | 0.484 |
| Hepatic arterial injection | Baseline | 50.1% | 0.111 | -0.21 | [-0.60,0.18] | 1.05 | 0.294 |
|  | 24 week | 0.0% | 0.469 | -0.11 | [-0.52,0.31] | 0.50 | 0.616 |
|  | Overall | 8.0% | 0.368 | -0.14 | [-0.30,0.03] | 1.65 | 0.098 |
| **Cell type** |  |  |  |  |  |  |  |
| BM-MSC | Baseline | 45.2% | 0.078 | -0.16 | [-0.44, 0.12] | 1.12 | 0.263 |
|  | 2 week | 57.8% | 0.124 | -0.34 | [-0.88, 0.19] | 1.26 | 0.209 |
|  | 4 week | 83.3% | 0.003 | -0.12 | [-0.72, 0.49] | 0.38 | 0.706 |
|  | **12 week** | **0.0%** | **0.939** | **-0.86** | **[-1.41, -0.32]** | **3.12** | **0.002** |
|  | 24 week | 79.0% | 0.001 | -0.18 | [-0.79, 0.43] | 0.58 | 0.563 |
|  | Overall | 65.3% | 0.000 | -0.21 | [-0.42, 0.00] | 1.98 | 0.048 |
| UC-MSC | Baseline | 96.2% | 0.000 | 0.27 | [-0.01, 0.55] | 1.41 | 0.158 |
|  | 12 week | 0.0% | 0.435 | -0.05 | [-0.27,0.17] | 0.44 | 0.659 |
|  | 24 week | 71.9% | 0.059 | 0.05 | [-0.58,0.68] | 0.16 | 0.873 |
|  | 48 week | 72.5% | 0.056 | -0.23 | [-0.87,0.42] | 0.69 | 0.489 |
|  | Overall | 92.3% | 0.000 | 0.43 | [-0.01,0.86] | 1.96 | 0.054 |
| **Times of treatment** |  |  |  |  |  |  |  |
| Single | Baseline | 35.1% | 0.187 | -0.01 | [-0.23, 0.21] | 1.28 | 0.199 |
|  | 2 week | 57.8% | 0.124 | -0.34 | [-0.88,0.19] | 1.26 | 0.209 |
|  | 4 week | 65.9% | 0.087 | -0.41 | [-1.01, 0.20] | 1.31 | 0.190 |
|  | **12 week** | **0.0%** | **0.939** | **-0.86** | **[-1.41,-0.32]** | **3.12** | **0.002** |
|  | **24 week** | **37.9%** | **0.200** | **-0.62** | **[-1.17, -0.08]** | **2.24** | **0.025** |
|  | Overall | 44.6% | 0.032 | -0.37 | [-0.57, -0.17] | 3.55 | 0.000 |
| Multiple | Baseline | 92.5% | 0.000 | 0.54 | [-0.25,1.34] | 1.33 | 0.183 |
|  | 4 week | 97.5% | 0.000 | 1.82 | [-0.91,4.54] | 1.31 | 0.191 |
|  | 12 week | 0.0% | 0.435 | -0.05 | [-0.27,0.17] | 0.44 | 0.659 |
|  | 24 week | 42.5% | 0.156 | 0.25 | [-0.04,0.54] | 1.70 | 0.089 |
|  | 48 week | 72.5% | 0.056 | -0.23 | [-0.87,0.42] | 0.69 | 0.489 |
|  | Overall | 88.8% | 0.000 | 0.34 | [0.02,0.65] | 2.11 | 0.035 |
| **Etiology** |  |  |  |  |  |  |  |
| HBV | Baseline | 92.0% | 0.000 | 0.61 | [-0.02, 1.23] | 1.91 | 0.056 |
|  | 4 week | 96.8% | 0.000 | 1.12 | [-0.31,2.56] | 1.54 | 0.124 |
|  | 12 week | 24.8% | 0.263 | -0.13 | [-0.42, 0.15] | 0.91 | 0.361 |
|  | 24 week | 57.0% | 0.073 | 0.17 | [-0.19,0.53] | 0.91 | 0.364 |
|  | 48 week | 56.5% | 0.100 | -0.09 | [-0.59, 0.42] | 0.34 | 0.736 |
|  | Overall | 87.0% | 0.000 | 0.25 | [-0.00, 0.51] | 1.93 | 0.053 |

**Additional file 1: Table S6.** Results of sensitivity analyses of the effect of MSCs therapy on TBIL level.

|  | **Random-effects model (Inverse-Variance)** | | | | |
| --- | --- | --- | --- | --- | --- |
| **TBIL at 4 week** | **SMD 95%-CI** | ***Z*** | **p-value** | ***I2*** | ***p*** |
| Pooled estimate | 0.65[-0.54,1.84] | 1.08 | 0.282 | 95.9% | 0.000 |
| Omitting 2011 Peng L | 0.95[-0.94,2.84] | 0.98 | 0.326 | 96.8% | 0.000 |
| Omitting 2014 Salama H | 1.12[-0.31,2.56] | 1.54 | 0.214 | 96.8% | 0.000 |
| Omitting 2017 Lin BL | 0.74[-1.22,2.71] | 0.74 | 0.459 | 97.2% | 0.000 |
| Omitting 2019 Xu WX | -0.12[-0.72,0.49] | 0.38 | 0.706 | 83.3% | 0.003 |
| **TBIL at 24 week** | **SMD 95%-CI** | ***Z*** | **p-value** | ***I2*** | ***p*** |
| Pooled estimate | -0.09 [-0.49, 0.31] | 0.45 | 0.649 | 74.7% | 0.001 |
| Omitting 2011 Peng L | -0.07[-0.49, 0.36] | 0.30 | 0.765 | 77.9% | 0.000 |
| Omitting 2012 Zhang Z | -0.06[-0.50, 0.38] | 0.25 | 0.801 | 76.6% | 0.001 |
| **Omitting 2014 Salama H** | **0.12[-0.17,0.41]** | **0.80** | **0.421** | **47.9%** | **0.088** |
| Omitting 2016 Suk KT | -0.15[-0.61,0.31] | 0.64 | 0.525 | 78.8% | 0.000 |
| Omitting 2016 Suk KT | -0.07[-0.51,0.38] | 0.29 | 0.772 | 77.2% | 0.001 |
| Omitting 2017 Lin BL | -0.22[-0.69,0.24] | 0.95 | 0.340 | 73.4% | 0.002 |
| Omitting 2021 Shi M | -0.20[-0.71,0.31] | 0.77 | 0.439 | 75.2% | 0.001 |
