# Supplementary material for: The assessment of mesenchymal stem cells therapy in acute on chronic liver failure and chronic liver disease: a systematic review and meta-analysis of randomized controlled clinical trials
Source: Stem Cell Res Ther. 2022 May 16;13:204. doi: 10.1186/s13287-022-02882-4 (PMC9109309; doi:10.1186/s13287-022-02882-4)
Supplement: Supplementary file 2 — Additional file 2: Figure S1. Funnel plots of MELD score at 24 weeks; ALB levels at 12 weeks, 24 weeks, and 48 weeks; TBIL at 4 weeks and 24 weeks; No asymmetry was observed in the funnel plots. No publication bias was found using Egger’s and Begg’s test. [file 13287_2022_2882_MOESM2_ESM.docx]

**Additional file 2: Figure S1**

**
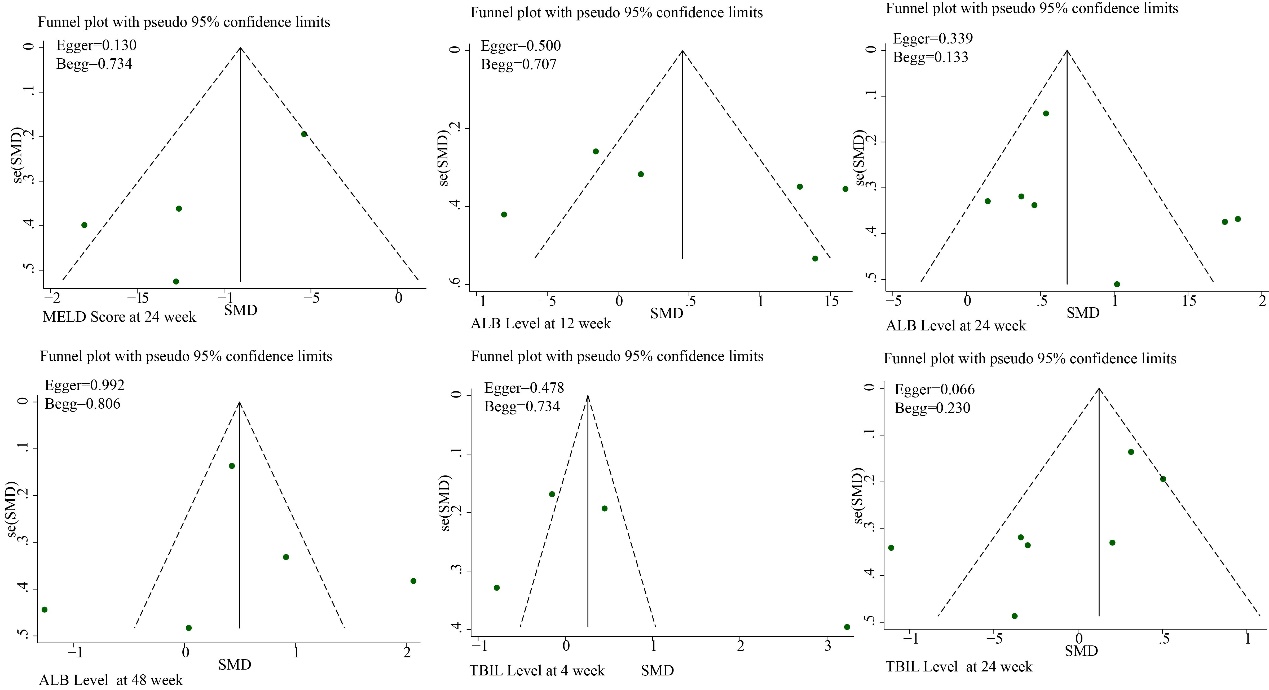
**

Funnel plots of MELD score at 24 weeks; ALB levels at 12 weeks, 24 weeks, and 48 weeks; TBIL at 4 weeks and 24 weeks; No asymmetry was observed in the funnel plots. No publication bias was found using Egger’s and Begg’s test.
